# Branched Endovascular Aneurysm Repair (BEVAR) to Rescue Failed Complex EVAR (C-EVAR): Technical Challenges and Outcomes in a 12-Case Series

**DOI:** 10.3390/jcm15103888

**Published:** 2026-05-18

**Authors:** Marco Virgilio Usai, Blanca Expósito-Camacho, Philipp Franke, Imam T. P. Ritonga, Jorge Balaguer-Germán, Martin J. Austermann

**Affiliations:** 1 Department of Vascular and Endovascular Surgery, St. Franziskus-Hospital, 48145 Münster, Germany; philipp.franke@sfh-muenster.de (P.F.); imam.ritonga@sfh-muenster.de (I.T.P.R.); martin.austermann@sfh-muenster.de (M.J.A.); 2 Department of Vascular and Endovascular Surgery, Fundación Alcorcón University Hospital, 28922 Madrid, Spain; b.expositocamacho@gmail.com; 3 Department of Cardiology, Puerta de Hierro University Hospital, 28035 Madrid, Spain; jjbg71@sescam.jccm.es

**Keywords:** branched/fenestrated endovascular aneurysm repair (F/BEVAR), chimney EVAR (ChEVAR), failed endovascular repair, type IA endoleak, thoracoabdominal aneurysm, reintervention

## Abstract

**Background:** Complex EVAR is a well-established option for treating complex aortic pathologies. However, depending on the type of it, long-term effectiveness is often compromised. For example, chimney EVAR is related to type IA endoleaks related to the gutter and proximal neck degeneration, late failures after fenestrated or branched EVAR are rare. Although redo-endovascular procedures are recommended for failed repairs, the use of branched endoprostheses (BEVAR) to address failed Complex EVAR (C-EVAR) cases is rarely documented. This study aims to evaluate the technical feasibility and 30-day outcomes of using BEVAR as a definitive rescue strategy for these patients. **Methods:** A retrospective single-center analysis was conducted on a series of twelve patients who had previously undergone failed C-EVAR. Clinical and procedure-related variables were collected. Statistical analysis was performed using Stata v18.0 software. **Results:** The reasons for reintervention were type Ia endoleak (ten patients), type Ib (one patient), and type III + Ia (one patient). Branched devices were used: eleven patients received the Zenith t-Branch (Cook Medical, Bloomington, IN, USA), and one received the G-Branch device (Lifetech Scientific, Shenzhen, China). Technical and clinical success was achieved in 11 out of 12 patients (91.7%). One perioperative death (due to haemothorax and sepsis) and three major complications were recorded in the first 30 days following repair. No patient of this cohort was deemed fit enough for open conversion. Imaging follow-up at 30 days revealed two type I leaks and seven type II leaks, with no type III leaks recorded. Patency was maintained in all treated visceral vessels (the celiac trunk, the superior mesenteric artery, and the renal arteries) in survivors. **Conclusions:** Repairing failed C-EVAR using branched endovascular aneurysm repair is a feasible and effective technique. This approach can resolve complex issues such as proximal sealing and component integrity failures, successfully excluding the aneurysmal sac while avoiding the morbidity and mortality associated with open surgery in high-risk patients.

## 1. Introduction

Complex EVAR (C-EVAR) including Ch-EVAR, FEVAR and BEVAR is a valuable tool for treating juxtarenal aortic aneurysms (JAAA). However, with Ch-EVAR, its long-term effectiveness is reduced by the occurrence of type IA endoleaks, which are associated with gutter formation or degeneration of the proximal aortic neck [1]. Repairing a failed C-EVAR procedure poses a significant surgical challenge. Simple proximal extensions can cause endoleaks to persist and converting to open surgery poses an unacceptable risk of morbidity and mortality for high-risk patients [2,3,4,5].

In this context, branched endoprostheses (BEVAR) are an advanced alternative for realigning the visceral segment. These devices offer several key advantages: they enable cannulation via a brachial approach, which is ideal for the cephalic configuration of previous bridging stents, and they provide superior mechanical overlap of at least 2 cm between the components, thereby minimizing the risk of new endoleaks [6].

Despite its potential, the scientific literature on rescuing complex repairs using a new branched device is extremely limited. While extensive series of sub-renal EVAR rescues using F/BEVAR have been published [7,8], reports of BEVAR following failed ChEVAR are limited to isolated cases [1].

This study aims to report on the technical feasibility and clinical outcomes using BEVAR to rescue failed C-EVAR.

## 2. Methods

### 2.1. Study Design and Population

A retrospective observational analysis was conducted on a consecutive series of twelve patients treated at a single centre between July 2023 and January 2026 using branched endovascular repair (BEVAR) following the failure of a previous C-EVAR procedure.

Reintervention was indicated by loss of proximal seal, presenting either as an isolated endoleak or in combination with other endoleaks.

This study complies with the Declaration of Helsinki. Local regulatory approvals were waived due to the retrospective nature of the study and the absence of patient-identifiable details shared between collaborators.

### 2.2. Stent Graft Planning and Design

All patients underwent preoperative thin-slice (1 mm) CT angiography with center-line reconstructions. The aim of the planning was to realign the visceral segment by establishing a new, definitive proximal sealing zone in the healthy aorta that was at least 20 mm long.

Branched devices such as Zenith t-Branch or custom-made branched devices (Cook Medical, Bloomington, IN, USA) and G-Branch (Lifetech Scientific, Shenzhen, China) were used.

The choice of device was driven by anatomical and clinical factors. For non-symptomatic or acute cases, custom-made devices with shorter proximal sealing or a smaller profile were planned to reduce the risk of spinal cord ischaemia due to excessive coverage of the intercostal arteries, and to facilitate navigation through tortuous iliac vessels. In cases involving chimney stents oriented towards the back of the aorta, off-the-shelf devices were not useful; therefore, a custom-made device was also used in these cases. For acute cases or anatomies treatable with an off-the-shelf graft configuration, this option was chosen.

### 2.3. Rescue Protocol

In cases of failed complex graft, the choice of BEVAR as the rescue method was driven by the fact that this technique allows for downward cannulation of the graft, which is easier in all clinical scenarios, including failed FEVAR, BEVAR, or ChEVAR. This study did not include other clinical situations of rescue, including Type Ia endoleak after standard EVAR. Nevertheless, the authors still prefer the use of BEVAR as a rescue technique in these situations.

### 2.4. Surgical Procedure

The surgical procedures were conducted in a hybrid operating theatre, with the patient receiving general anaesthesia.

A left axillary surgical approach and at least one percutaneous femoral access were routinely employed. Haemostasia was achieved using the Prostar XL percutaneous vascular surgical system (Abbott Vascular Inc., Redwood City, CA, USA).

The visceral bypass technique was standardised in order to navigate around pre-existing metallic components, primarily using a combination of balloon-expandable and self-expanding stents to ensure flexibility and sealing.

It is evident that prophylactic cerebrospinal fluid drainage was not performed in any of the cases within the series.

### 2.5. Definitions and Follow-Up

Technical success: The successful deployment of the device, with patency of the target vessels and no endoleaks evident on the final follow-up angiography.

Clinical success: Survival beyond 30 days, effective exclusion of the aneurysmal sac, and no device-related reinterventions in the perioperative period. Type II endoleaks in the completion angiography were no considered a technical or clinical failure, but an expected finding after endovascular treatment. Authors consider Type II endoleaks of relevance only in the case of causing factor for sack growth.

### 2.6. Statistical Analysis

Statistical analysis was performed using Stata version 18.0 (StataCorp LLC, College Station, TX, USA) software.

Continuous variables were summarised as the mean ± SD or the median (IQR) and compared using a Student’s *t*-test or a Mann–Whitney U-test. A paired Student’s *t*-test or Wilcoxon signed-rank test was used. Categorical variables were depicted as percentages of the total group and compared using either a chi-squared test or Fisher’s exact test. We performed a multivariable logistic regression analysis to identify predictors of the composite endpoint. A two-sided *p* value of less than 0.05 was considered statistically significant.

## 3. Results

### 3.1. Population Characteristics and Clinical Profile

The cohort comprised 12 patients, with a marked predominance of males (83.3%). The average age of the patients was 78 years (SD ± 6.7). The patients exhibited significant systemic morbidity with 91.7% diagnosed as hypertensive and undergoing treatment with a single antihypertensive agent. Additionally, 33.3% were identified as diabetic, while 41.7% had been diagnosed with chronic kidney disease, with baseline creatinine levels recorded at 2.4 mg/dL. The majority of patients (83.3%) were being treated with a single antiplatelet agent; of the remaining two patients, one was receiving anticoagulant therapy and the other was not taking any such drugs (Table 1).

With regard to the classification of anaesthetic risk, the majority of the cohort (91.7%) were categorised as ASA 3, with a single patient (0.9%) classified as ASA 4.

### 3.2. Analysis of Initial ChEVAR Failure

At the time of the initial C-EVAR procedure, the aneurysm’s maximum mean diameter was 64.6 mm (SD ± 11.16).

The most commonly used device in previous interventions was the Endurant Stent Graft System (Medtronic Cardiovascular, Santa Rosa, CA, USA), which was used in ten cases (five with a cuff and five with a bifurcated configuration). This was followed by one case each of the Zenith Fenestrated AAA Endovascular Graft (Cook Medical, Bloomington, IN, USA) and the G-Branch (Lifetech Scientific, Shenzhen, China).

The complexity of the original C-EVAR procedure is reflected in the number of chimneys implanted: one in six patients, two in two patients, three in three patients and four in one patient. The covered stents used for the chimneys were predominantly Advanta V12 (Atrium Medical Corporation, Hudson, NH, USA) (91.7%), though one patient received a combination of Advanta and Gore Viabahn (W.L. Gore & Associates, Flagstaff, AZ, USA) (see Table 2).

### 3.3. BEVAR Rescue Procedure

When the BEVAR rescue procedure was deemed necessary, the primary cause of failure was a type Ia endoleak (83.3%), which, in one instance, resulted in the aneurysmal sac rupturing. Other indications included a combination of types Ia and Ib (8.3%), as well as a combination of types Ia and III (8.3%). At the time of reintervention, six patients had an aneurysmal sac larger than 8 cm and one patient was experiencing symptoms. The mean maximum diameter of the aneurysm at the time of BEVAR was 71.6 mm (SD ± 20.7).

One patient required a thoracic procedure, with subsequent treatment in two stages.

Realignment of the visceral segment was primarily performed using the Cook Zenith T-Branch device (Cook Medical, Bloomington, IN, USA) in 91.7% of cases, while one patient received a G-Branch device (Lifetech Scientific, Shenzhen, China). A greater proportion of the devices were custom-made (75%) than off-the-shelf (25%).

**Figure 1:**
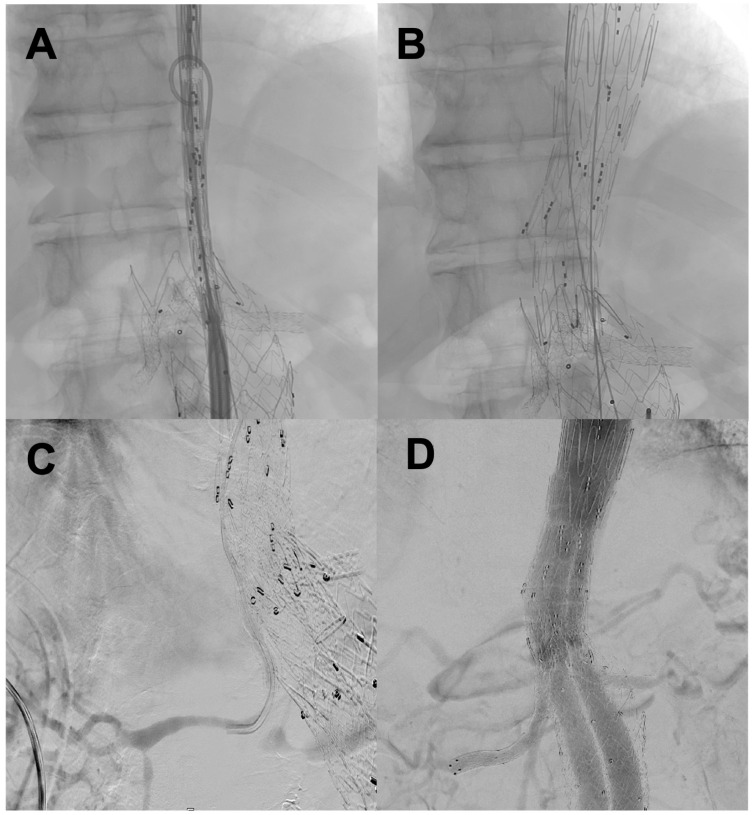
Intraoperative angiographic images: (**A**) Predeployment and positioning of the branched device; (**B**) Deployment of the branched device; (**C**) An example of cannulation of the target vessel with previous chimney stent (in this case, the right renal artery); (**D**) Final angiography.

Technical details of the implant (Figure 1):

- Anchorage sites: The predominant proximal site was Zone 5 (83.3%), with isolated cases in Zones 3 and 4. Distally, nine cases were sealed over previous iliac extensions (Table 3).

- Surgical access: All patients underwent anterograde cannulation of the visceral vessels via a left axillary surgical access. All patients underwent percutaneous femoral access; a bilateral femoral approach was required in seven patients. Percutaneous closure using the Prostar XL system (Abbott Vascular Inc., Redwood City, CA, USA) was performed in all cases. One patient required conversion to a surgical approach. This was due to failure of the closure device.
- Visceral management: The four main visceral vessels (the celiac trunk [CT], the superior mesenteric artery [SMA] and both renal arteries) were treated in ten patients. The visceral bypass technique was standardised through the combined use of viabahn balloon expandable stent (VBX) and Viabahn covered stents (W.L. Gore & Associates, Flagstaff, AZ, USA), particularly in the SMA (91.7%) and CT (83.3%). In the renal arteries, the most common approach was a combination of VBX and Viabahn stents (66.7%), although single stents (VBX or BeGraft Plus [Bentley InnoMed GmbH, Hechingen, Germany]) and triple combinations (VBX + Viabahn + BeGraft Plus) were also used (Table 4).

The median procedure duration was 226.5 min (IQR 81.5). The mean fluoroscopy time was 68.1 min (±24.6 SD), with a mean contrast volume of 178.5 mL (IQR 62.5) (Table 5).

Prophylactic cerebrospinal fluid drainage was not performed on any patient in this series.

### 3.4. Perioperative Outcomes (Up to 30 Days)

The average length of stay in the ICU was 1.9 days (SD ± 0.3).

Technical and clinical success was achieved in eleven out of twelve patients (91.7%). No conversion to open surgery was required during the rescue procedure. One perioperative death (8.3%) was recorded due to haemothorax and sepsis. This patient was treated for failed FEVAR. Following surgery, a thorax drainage was positioned due to symptomatic pleural effusion. Afterwards, active bleeding was observed due to the drainage being malpositioned, resulting in lung injury. This resulted in a rapidly evolving shock situation, which ended in death.

Furthermore, three major perioperative complications were identified: one exacerbation of chronic obstructive pulmonary disease (COPD) associated with a haematoma; one pneumothorax with haemothorax and sepsis; and one isolated haematoma. These perioperative complications were associated with bilateral percutaneous femoral access (Fisher’s exact test, *p* = 0.045).

At the 30-day imaging follow-up, seven type II and two type Ib endoleaks were detected, with no type III cases recorded (Table 6).

**Figure 2:**
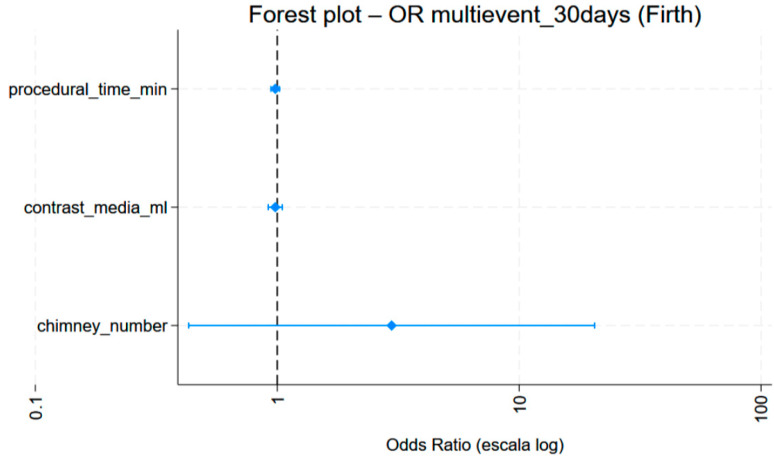
Penalized multivariate model (Firth), analysing procedure duration, contrast volume and number of previous chimneys.

No differences were observed in the risk of a perioperative event according to procedure duration (OR = 0.98; *p* = 0.106; 95% CI: 0.95–1.00). Similarly, no statistically significant difference was observed in the 30-day event risk in relation to the number of previous chimneys (OR = 2.46; *p* = 0.216; 95% CI: 0.59–10.18). Increasing the amount of contrast did not lower the probability of an event occurring (OR = 0.97; *p* = 0.146; 95% CI: 0.93–1.01). In the penalised multivariate model (Firth), no variable reached statistical significance. However, a non-significant trend towards a higher risk of events at 30 days was observed in patients with a higher number of chimneys (OR 2.97) (see Figure 2).

Notably, there were no spinal cord ischaemia events throughout the series.

### 3.5. Mid-Term Follow-Up and Reinterventions

Among survivors, 100% patency was maintained in the superior mesenteric artery, renal arteries and iliac branches. The celiac trunk remained patent in all cases except one, where there was an occlusion prior to BEVAR.

Late reinterventions were required in three patients due to the complexity of the residual aortic pathology.

- Persistent type Ia endoleak; Following a failed attempt at extension via TEVAR, the patient required a laparotomy with sac plication due to continued sac enlargement.
- Type Ib endoleak: Bilateral iliac extensions were required and, given the persistence of the leak, a right iliac branch endovascular device was finally implanted.
- Stent recoil: One patient required treatment for stent recoil in the right renal artery.

A second death occurred more than 30 days after the procedure, due to sepsis that was not related to the aneurysm. No deaths were directly related to the aneurysm during the study period (Table 7).

## 4. Discussion

The endovascular treatment of complications arising from complex aortic repair procedures is one of the most challenging scenarios in modern vascular surgery. Although the rescue of failed infrarenal stents using F/BEVAR has been well documented, with technical success rates exceeding 94% [2,3,5], the rescue of failed complex EVARs is rarely described in medical literature. To the best of our knowledge, our series of 12 cases is the largest reported experience of using branched devices to resolve C-EVAR failures, significantly exceeding existing case reports involving just one patient [1].

The durability of the CH-EVAR technique is compromised by the presence of gutters and degeneration of the aortic neck. These factors increase the risk of patients developing type IA endoleaks. In our series, the predominant indication for rescue surgery was type IA endoleak (in ten out of twelve patients), which is consistent with the findings reported by Dayan et al. An aneurysmal sac enlargement due to loss of proximal sealing necessitated complex reintervention in these cases [1]. Converting to open surgery for these patients would be extremely invasive, with morbidity and mortality rates that could be prohibitive [8]. Consequently, realignment of the visceral segment using BEVAR is considered the ultimate rescue strategy since it enables the new device to be secured in a healthy section of the aorta, typically closer to the proximal end (Zones 3–5) [1,9].

One of the greatest technical challenges of the procedure is cannulating the visceral vessels through the multiple layers of metal of the existing stents [8,10]. Furthermore, while the position of the target stents is quite standard in previous FEVAR and BEVAR procedures, in Ch-EVAR the position of the stents may be more challenging. In two patients in our cohort, the renal stents were oriented towards the dorsal part of the aorta, necessitating the use of a custom-made device with branches at the 6 o’clock position to enable cannulation. The widespread use of the Cook Zenith T-Branch device (Cook Medical, Bloomington, IN, USA) in our cohort (91.7% of patients) and the inner branch design (in 75% of customised devices) was key. As Silverberg et al. and Dayan et al. point out, inner branches offer several advantages. They provide superior mechanical overlap with the bridge stent (up to 2 cm), which minimises the risk of type III endoleaks. They also enable deployment in narrow aortas (from a minimum of 18 mm), which outer branches would not be able to expand into [1,6].

Furthermore, its design is ideal for anterograde cannulation via an axillary or brachial approach. This is necessary when the previous chimney stents point upwards [1].

Our technical and clinical success rate of 91.7% is comparable to that of large-scale FEVAR rescue studies (83–94%) [9]. Notably, despite the high complexity and extensive aortic coverage, we did not record any cases of perioperative spinal cord ischemia. According to the literature, prior repair may promote the development of a collateral network that protects the spinal cord during a second procedure. However, spinal ischaemia remains a potential concern with these procedures, with rates of up to 2.4% reported in systematic reviews of complex rescue procedures. The perioperative mortality rate in our study population (8.3%, with one case unrelated to the aneurysm) is within the expected range for this frail, high-risk group [8].

A key finding of our study is that re-operating on previously manipulated anatomical structures does not appear to directly correlate with an increase in perioperative morbidity and mortality. Our analysis revealed no significant difference in the risk of a perioperative event based on procedure duration (OR = 0.98; *p* = 0.106; 95% CI: 0.95–1.00). This is particularly relevant given that, as reported by Taher et al. (301 min vs 253 min) and Schanzer et al. (5.2 h vs 4.6 h), complex aortic rescue procedures typically require significantly longer operative times than primary repairs [7,11].

Furthermore, prior metallic burden, represented by the number of chimney stents, was not a decisive predictor of short-term failure, despite hindering visibility of the structures. There was no statistically significant difference in the risk of an event at 30 days in relation to the number of prior stents (OR = 2.46; *p* = 0.216; 95% CI: 0.59–10.18), nor was a greater amount of contrast associated with a lower probability of an event (OR = 0.97; *p* = 0.146; 95% CI: 0.93–1.01). These results suggest that, once the learning curve has been overcome and advanced imaging technologies are in place, surgeons can manage the technical complexity of these procedures without compromising patient safety. It should be noted that all of the surgeons who performed the procedures are high-volume practitioners, performing 20–50 B/FEVAR procedures per year.

It is important to note that although no variable reached statistical significance in the penalised multivariate Firth model, a non-significant trend towards a higher risk of events at 30 days was identified in patients with a higher number of chimneys (OR 2.97). While this trend is inconclusive due to the size of our sample, it underscores the need for extremely meticulous planning when dealing with multi-axial visceral realignments.

The concept of endovascular repair as the management of a ‘chronic condition’ is evident in our series, in which 25% of patients required late reinterventions to maintain aneurysm exclusion. This percentage is consistent with that reported by Dossabhoy et al. and Zettervall et al. in their analyses of reinterventions following F/BEVAR (25–26%) [12,13]. The need to perform a laparotomy with sac plication on a patient with a persistent Ia endoleak shows that, although the endovascular approach is preferred, surgeons must be prepared to perform more conservative open rescue procedures if endovascular sealing fails [10].

Conversely, the majority of patients were initially treated with Ch-EVAR due to their limited life expectancy. In this regard, BEVAR demonstrates superior long-term outcomes in comparison to Ch-EVAR [7].

This study has limitations inherent in its retrospective design and sample size, although it is the largest series available to date. The variability in the bypass stents used reflects the absence of a device designed specifically for this ‘stent-in-stent’ scenario [12]. Furthermore, the average follow-up period is too short to evaluate the long-term durability of internal branches in aortas that have previously been manipulated. Moreover, the multivariable analysis is underpowered, and conclusions about predictive factors should be interpreted with caution.

## 5. Conclusions

In conclusion, rescuing a failed C-EVAR with BEVAR appears to be feasible and effective technique in this small cohort. Our series shows that branched devices, combined with careful planning and advanced imaging, can achieve a durable seal and maintain visceral patency.

Further multicentre studies with longer follow-up periods are required to establish this strategy as the standard of care for C-EVAR.

## Figures and Tables

**Table 1:** Demographics and baseline characteristics.

Variable	Result
Age (years), mean ± SD	78 ± 6.7
Male sex, *n* (%)	10 (83.3%)
Female sex, *n* (%)	2 (16.7%)
Hypertension, *n* (%)	11 (91.7%)
Diabetes mellitus type II, *n* (%)	4 (33.3%)
Renal function	
Creatinine > 2.4 mg/dL, *n* (%)	5 (41.7%)
Normal renal function, *n* (%)	7 (58.3%)
Moderate lung disease, *n* (%)	2 (16.7%)
Moderate heart disease, *n* (%)	1 (8.3%)
Antithrombotic therapy	
Single antiplatelet, *n* (%)	10 (83.3%)
Anticoagulation, *n* (%)	1 (8.3%)
No antithrombotic therapy, *n* (%)	1 (8.3%)

**Table 2:** Previous ChEVAR procedure characteristics, chimney endovascular aortic repair (ChEVAR), branched endovascular aortic repair (BEVAR).

Variable	Result
Aneurysm diameter at ChEVAR (mm), mean ± SD	64.6 ± 11.16
Number of chimneys	
1 chimney, *n* (%)	6 (50.0%)
2 chimneys, *n* (%)	2 (16.7%)
3 chimneys, *n* (%)	3 (25.0%)
4 chimneys, *n* (%)	1 (8.3%)
Previous endoprosthesis	
Endurant (cuff + bifurcated), *n* (%)	10 (83.3%)
FEVAR Cook, *n* (%)	1 (8.3%)
BEVAR G Branch, *n* (%)	1 (8.3%)
Chimney stents used	
Advanta only, *n* (%)	11 (91.7%)
Advanta + Viabahn, *n* (%)	1 (8.3%)

**Table 3:** BEVAR indications and procedural details. American Association Anesthesiology (ASA), Branched endovascular aortic repair (BEVAR).

Variable	Result
Reintervention indication	
Type Ia endoleak, *n* (%)	10 (83.3%)
Including sack rupture, *n*	1
Type Ia + Ib endoleak, *n* (%)	1 (8.3%)
Type Ia + III endoleak, *n* (%)	1 (8.3%)
ASA classification	
ASA 3, *n* (%)	11 (91.7%)
ASA 4, *n* (%)	1 (8.3%)
Urgency status	
Elective, *n* (%)	5 (41.7%)
Sack diameter >8 cm, *n* (%)	6 (50.0%)
Symptomatic, *n* (%)	1 (8.3%)
Aneurysm diameter at BEVAR (mm), mean ± SD	71.6 ± 20.7
Proximal landing zone	
Zone 3, *n* (%)	1 (8.3%)
Zone 4, *n* (%)	1 (8.3%)
Zone 5, *n* (%)	10 (83.3%)
Distal landing zone	
Previous main body, *n* (%)	3 (25.0%)
Previous iliac extensions, *n* (%)	9 (75.0%)

**Table 4:** Stent configurations by visceral vessel. viabahn balloon expandable stent (VBX).

Variable	Result
Celiac trunk	
VBX alone, *n* (%)	1 (8.3%)
VBX + Viabahn, *n* (%)	10 (83.3%)
Advanta + Plug, *n* (%)	1 (8.3%)
Superior mesenteric artery	
VBX alone, *n* (%)	1 (8.3%)
VBX + Viabahn, *n* (%)	11 (91.7%)
Right renal artery	
VBX alone, *n* (%)	1 (8.3%)
VBX + Viabahn, *n* (%)	7 (58.3%)
VBX + Viabahn + BeGraftPlus, *n* (%)	1 (8.3%)
BeGraft + Viabahn, *n* (%)	1 (8.3%)
BeGraftPlus + VBX, *n* (%)	1 (8.3%)
Viabahn + Advanta, *n* (%)	1 (8.3%)
Left renal artery	
VBX + Viabahn, *n* (%)	8 (66.7%)
VBX + Viabahn + BeGraftPlus, *n* (%)	1 (8.3%)
Advanta + iCover, *n* (%)	1 (8.3%)
Advanta + Viabahn, *n* (%)	1 (8.3%)

**Table 5:** Surgical technique and operative data. Superior mesenteric artery (SMA).

Variable	Result
BEVAR endoprosthesis	
Cook t-Branch, *n* (%)	11 (91.7%)
Lifetech G-Branch, *n* (%)	1 (8.3%)
Device type	
Off-the-shelf, *n* (%)	3 (25.0%)
Custom-made, *n* (%)	9 (75.0%)
Sheath sizes (F)	16, 18, 20, 22, 24
Distal configuration	
Bifurcated, *n* (%)	8 (66.7%)
Straight tube, *n* (%)	4 (33.3%)
Visceral vessel configuration	
Celiac + SMA + both renal, *n* (%)	10 (83.3%)
Celiac + SMA + right renal, *n* (%)	1 (8.3%)
SMA + both renal, *n* (%)	1 (8.3%)
Visceral vessel access	
External antegrade, *n* (%)	11 (91.7%)
Fenestration (right renal), *n* (%)	1 (8.3%)
Prophylactic CSF drainage, *n* (%)	0 (0%)
Procedure time (min), median (IQR)	226.5 (81.5)
Fluoroscopy time (min), mean ± SD	68.1 ± 24.6
Contrast volume (mL), median (IQR)	178.5 (62.5)

**Table 6:** Perioperative outcomes.

Variable	Result
ICU stay (days), mean ± SD	1.9 ± 0.3
ICU stay range (days)	1–2
Perioperative mortality, *n* (%)	1 (8.3%)
Conversion to open surgery, *n* (%)	0 (0%)
Technical success, *n*/N (%)	11/12 (91.7%)
Clinical success, *n*/N (%)	11/12 (91.7%)
Access complications	
Percutaneous to surgical conversion, *n* (%)	1 (8.3%)
Spinal cord ischemia, *n* (%)	0 (0%)
30-day endoleaks	
Type I, *n* (%)	2 (16.7%)
Type II, *n* (%)	7 (58.3%)
Type III, *n* (%)	0 (0%)
Perioperative complications	
COPD exacerbation + hematoma, *n* (%)	1 (8.3%)
Pneumothorax + hemothorax + sepsis, *n* (%)	1 (8.3%)
Hematoma, *n* (%)	1 (8.3%)

**Table 7:** Follow-up and reintervention outcomes. Thoraci endovascular aortic repair (TEVAR).

Variable	Result
Aneurysm-related mortality, *n* (%)	0 (0%)
Total deaths, *n* (%)	2 (16.7%)
Early death (<30 days), *n* (%)	1 (8.3%)
Cause: hemothorax + sepsis	
Late death (>30 days), *n* (%)	1 (8.3%)
Cause: sepsis	
Reinterventions required	
One reintervention, *n* (%)	3 (25.0%)
Two reinterventions, *n* (%)	2 (16.7%)
Reintervention details	
Type Ia, TEVAR, laparotomy/sac plicature, *n* (%)	1 (8.3%)
Right renal stent recoil, n (%)	1 (8.3%)
Type Ib, bilateral iliac or right iliac branch, *n* (%)	1 (8.3%)
Vessel patency at follow-up	
Celiac trunk (%), *n*	100%, 11
(pre-existing occlusion)	
Superior mesenteric artery (%), *n*	100%, 12
Renal arteries (%), *n*	100%, 24
Iliac branches (%), *n*	100%, 16

## Data Availability

The presented data is available on request from the corresponding author.
